# Impact of extubation failure on the duration of mechanical ventilation in the pediatric population

**DOI:** 10.31744/einstein_journal/2025AO0705

**Published:** 2025-04-25

**Authors:** Thamires de Carvalho Silvério, Cristiane do Prado, Milena Siciliano Nascimento

**Affiliations:** 1 Hospital Israelita Albert Einstein São Paulo SP Brazil Hospital Israelita Albert Einstein, São Paulo, SP, Brazil.

**Keywords:** Airway extubation, Respiration, artificial, Ventilator weaning, Intensive care units, Pediatric, Child

## Abstract

**Objective:**

To compare the epidemiological profiles of patients who experienced extubation failure with those who achieved success and to evaluate the impact of extubation failure on total mechanical ventilation duration.

**Methods:**

An observational study with both retrospective and prospective components was conducted on patients admitted to the Pediatric Intensive Care Unit of a private hospital. This study included patients who underwent mechanical ventilation and were extubated between January 2017 and December 2022. Patients were classified into extubation Success or Failure Groups, with failure defined as requiring a return to invasive mechanical ventilation within 48hs post-extubation. Epidemiological factors, including age, pre-existing illnesses, and time on invasive mechanical ventilation, were analyzed.

**Results:**

A total of 173 patients were included, of which 9 (5.2%) required reintubation within 48hs. The total duration of mechanical ventilation differed significantly between the Success Group (3 days [1.8; 6.6]) and the Failure Group (6.5 days [5.6; 9.3]), p=0.004. Upper airway obstruction was identified as the primary cause of extubation failure.

**Conclusion:**

This study demonstrated that extubation failure doubled the total duration of mechanical ventilation compared to successful extubation. These findings highlight the importance of daily patient assessment with clearly defined clinical criteria to ensure mechanical ventilation is discontinued precisely when the patient is ready-neither prematurely nor excessively delayed.

## In Brief

The study demonstrated that extubation failure prolonged the total duration of mechanical ventilation to twice that observed in patients with successful extubation. These findings emphasize the critical need for daily patient assessment guided by well-defined clinical criteria to ensure mechanical ventilation is discontinued precisely when the patient is clinically prepared-neither prematurely nor unnecessarily delayed.

## Highlights

■ In patients who experienced extubation failure, the total ventilation time was twice as long as that of patients with successful extubation.

■ The incidence of extubation failure was 5.2%, which is relatively low compared to other studies.

■ The study revealed no significant association between the presence of pre-existing illnesses and extubation outcomes.

■ Results indicated that the primary cause of extubation failure was upper airway obstruction, accounting for 67% of cases requiring reintubation.

## Graphical abstract

**Figure:**
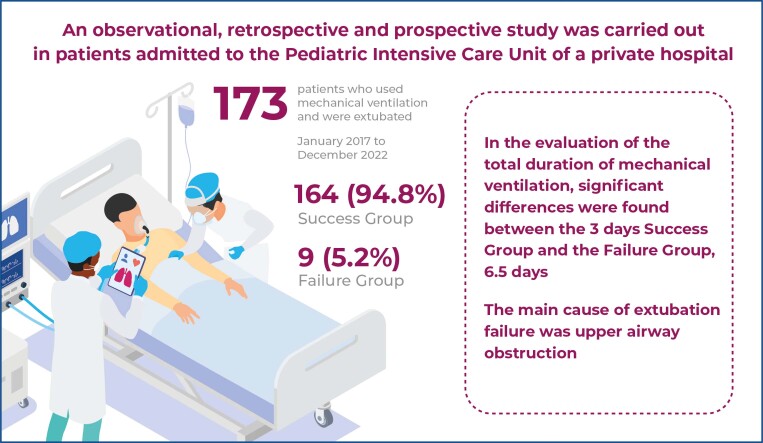

## INTRODUCTION

Invasive mechanical ventilation (IMV) is a common intervention in pediatric intensive care units (P-ICUs). Studies report that up to 63% of pediatric patients admitted to P-ICUs with acute respiratory illnesses require mechanical ventilation.^1^

While IMV is a life-saving intervention that improves gas exchange and reduces the work of breathing, it is associated with risks such as mechanical ventilation-induced lung injury,^2^ ventilator-associated pneumonia (VAP),^3^ hemodynamic alterations,^4^ upper airway obstruction (UAO), and diaphragmatic atrophy.^5^

Since many complications are linked to the duration of mechanical ventilation, extubation should be performed as early as clinically feasible.^3^ However, premature extubation without adequate clinical readiness may result in extubation failure.

Daily patient assessments are essential to determine clinical readiness for extubation and, when conducted effectively, can reduce the duration of mechanical ventilation.^6^ These assessments evaluate multiple factors, including sedation level, hemodynamic stability, fluid balance, ventilatory and gasometric parameters, cough effectiveness, and inspiratory muscle function.^7^ Alongside thorough clinical evaluations, the spontaneous breathing test can support decision-making regarding extubation readiness.^8,9^

The incidence of extubation failure in the literature ranges from 2.7% to 22%,^6^ with studies showing a significant association between failure and increased mortality.^10,11^ Given the considerable impact of extubation failure on patient outcomes, monitoring its occurrence is now a key indicator of care quality.^7^ However, a failure rate near zero, accompanied by prolonged mechanical ventilation times, does not necessarily signify high-quality care. Both metrics must be evaluated together to provide a complete picture of care quality.

While the definitions of extubation failure and eligible patient populations for this indicator are well-established in this literature,^12^ consensus on how to calculate the duration of mechanical ventilation remains elusive in adults^13^ and was only recently established in pediatrics.^7^

In most studies, the duration of mechanical ventilation is typically considered only for the first phase of ventilation. When patients fail and require reintubation, this second phase is often treated as an independent event. Some authors advocate for defining the duration of mechanical ventilation as the entire period from the initiation of IMV until its successful withdrawal, with the time from both phases combined in cases of failure.^14^

## OBJECTIVE

To compare the epidemiological profiles of patients who experienced extubation failure with those who achieved successful extubation and to evaluate the impact of extubation failure on the total duration of mechanical ventilation.

## METHODS

### Type and place of study

An observational study with both retrospective and prospective components was conducted. The retrospective analysis utilized an anonymized database covering the period from January 2017 to February 2022. Prospective data were collected between March and December 2022 in the Pediatric Intensive Care Unit of a private hospital.

### Ethical aspects

The project was submitted to and approved by the Research Ethics Committee of *Hospital Israelita Albert Einstein* (CAAE: 54459021.2.0000.0071; # 6.124.950). For retrospective data, the requirement for a Free and Informed Consent Form was waived. For the prospective study, after approval by the Research Ethics Committee, the parents or legal guardians of patients meeting the inclusion criteria provided written informed consent. This study adheres to the recently amended Declaration of Helsinki (1995).

### Inclusion and exclusion criteria

Children under 18 years of age who required orotracheal intubation and mechanical ventilation for more than 24hs and were subsequently extubated were included.

Patients were excluded if they remained on mechanical ventilation for less than 24hs, were not extubated due to death or tracheostomy, or had insufficient data for analysis.

### Data collect

All study data were retrieved from an institutional database and entered into a REDCap (Research Electronic Data Capture) database by an independent research assistant from the maternal and child department, who was not involved as an author of this study. The data provided to the authors were fully anonymized.

Patients were categorized into extubation Success and Failure Groups, with failure defined as the need to return to invasive mechanical ventilation within 48hs post-extubation.

### Variables analyzed

To categorize the population and compare groups with successful versus failed extubation, demographic variables were collected, including age (months), gender (female or male), weight (kilograms), pre-existing illnesses, patient profile (clinical or surgical), and cause of intubation. Intubation causes included worsening lung illness, altered level of consciousness, convulsive syndrome, apnea, hypersecretion, surgical procedure, cardiorespiratory arrest, hemodynamic instability, UAO, and acute respiratory failure. Additional factors analyzed included the need for non-invasive ventilation (NIV) before intubation and the duration of IMV. The duration of mechanical ventilation was calculated from the time of intubation to successful weaning from ventilation. For patients requiring reintubation within 48hs, the reintubation duration was added to the initial ventilation time to determine the total ventilation duration.

### Planning of statistical methods

The sample was characterized using the mean and standard deviation, minimum and maximum values, median, and quartiles for quantitative variables, and by absolute and relative frequencies for qualitative variables.

Comparisons of the characteristics related to extubation failure were conducted using the χ^2^ test or Fisher’s exact test for qualitative variables and the Mann-Whitney test for quantitative variables, based on the data distribution characteristics. Data normality was assessed using the Shapiro-Wilk test, boxplot charts, histograms, and quartile comparison charts.

All analyses were performed using SPSS, version 26.0, with a significance level set at 5%.

## RESULTS

The total sample consisted of 331 patients. The following were excluded: 102 patients who remained under invasive mechanical ventilation for less than 24hs, 40 whose outcome was tracheostomy or death, 5 who were older than 216 months (18 years), and 11 with insufficient data for analysis. This resulted in 158 patients who did not meet the predefined criteria.

**Figure 1:**
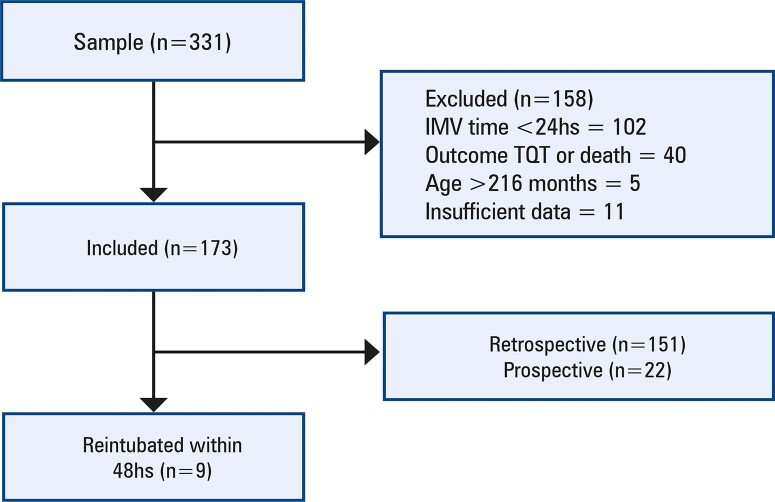
Patients selection process

A total of 173 patients were included in this study, comprising 151 retrospective inclusions and 22 prospective inclusions. Among them, 9 patients (5.2%) required reintubation within 48hs (Figure 1).

The study illustrates the inclusion and exclusion criteria applied to the initial sample (n=331), resulting in the final study population. A total of 158 patients were excluded, leaving 173 patients who met the criteria for inclusion. Of these, 151 patients were from the retrospective cohort, and 22 were from the prospective cohort. Nine patients (5.2%) required reintubated within 48hs of extubation.

Demographic characteristics and mechanical ventilation data are presented in table 1. These data include the total number of patients and comparisons between the extubation Success and Failure Groups. No significant differences were observed for the evaluated characteristics, except for total ventilation time: Success Group 3.0 days [1.8; 6.6] *versus* Failure Group 6.5 days [5.6; 9.3], p=0.004. Data are reported as medians with interquartile ranges.

**Table 1:** Comparison of characteristics of patients due to extubation failure

		Total (n=173) n (%)	Success (n=164) n (%)	Failure (n=9) n (%)	p value
Gender					0.605 ^†^
	Male	100 (57.8)	96 (58.5)	4 (44.4)	
	Female	73 (42.2)	68 (41.5)	5 (55.6)	
Age in months					0.197 ^£^
	Median [Quartiles]	16 [5; 72]	16 [5; 77]	5 [4; 25]	
	Mínimum - Maximum	0 - 215	0 - 215	1 - 126	
Pre-existing illnesses					>0.999^‡^
	Respiratory System	16 (17.6)	15 (17.4)	1 (20.0)	>0.999^‡^
	Neurological System	33 (36.3)	31 (36.0)	2 (40.0)	>0.999^‡^
	Cardiovascular System	18 (19.8)	17 (19.8)	1 (20.0)	>0.999^‡^
	Urinary system	5 (5.5)	5 (5.8)	0 (0)	>0.999^‡^
	Oncological	17 (18.7)	17 (19.8)	0 (0)	0.579^‡^
	Immunological system	4 (4.4)	4 (4.7)	0 (0)	>0.999^‡^
	Others	9 (9.9)	8 (9.3)	1 (20.0)	0.413^‡^
Indication of the IMV					0.468^‡^
	Exacerbation of lung illness	8 (4.6)	7 (4.3)	1 (11.1)	
	Alteration level of consciousness	12 (6.9)	12 (7.3)	0 (0)	
	Convulsive syndrome	9 (5.2)	9 (5.5)	0 (0)	
	Apnea	5 (2.9)	5 (3.0)	0 (0)	
	Surgical procedure	59 (34.1)	57 (34.8)	2 (22.2)	
	Cardiopulmonary arrest	8 (4.6)	7 (4.3)	1 (11.1)	
	Hemodynamic instability	6 (3.5)	5 (3.0)	1 (11.1)	
	Upper airway obstruction	2 (1.2)	2 (1.2)	0 (0)	
	Acute respiratory failure	64 (37.0)	60 (36.6)	4 (44.4)	
Time (days) on mechanical ventilation					0.720^£^
	Median [Quartiles]	3 [1.8; 6.2]	3 [1.8; 6.6]	3,1 [1.3; 5.4]	
	Minimum - Maximum	1 - 70	1 - 70	1.1 - 10	
Total time on mechanical ventilation					0.004^£^*
	Median [Quartiles]	3,2 [1.9; 6.6]	3 [1.8; 6.6]	6.5 [5.6; 9.3]	
	Minimum – Maximum	1 - 70	1 - 70	4.7 - 15.3	

^†^ χ^2^ test; ^‡^ Fisher exact test; ^£^ Mann-Whitney test: median [quartiles]; * Significant difference was found for total ventilation time: success 3.0 days [1.8; 6.6] and failure 6.5 days [5.6; 9.3], p=0.004.

Data are presented in medians and interquartile range and minimum and maximum for quantitative variables, and by absolute and relative frequencies, for qualitative variables; IMV: invasive mechanical ventilation.

Among the patients who required reintubation within 48hs, 6 cases (67%) were due to UAO, 2 cases (22%) were due to acute respiratory failure, and 1 case (11%) was due to hemodynamic instability.

## DISCUSSION

Our study compared pediatric patients with extubation failure and success and found a significant association between extubation failure and an increase in total mechanical ventilation time. The results demonstrated that patients who experienced extubation failure had a total ventilation time that was twice as long as that of patients with successful extubation.

The duration of IMV is a crucial outcome for assessing ventilation clearance in pediatrics and serves as a balancing measure for extubation failure. However, there is no consensus among studies regarding the measurement of mechanical ventilation time as a variable.^7,13-15^ Contentin et al highlighted the heterogeneity in measuring IMV duration, noting that it may be reported as only the first period of ventilation or as the sum of all ventilation periods, including those following reintubation.^13^

The method used to analyze this variable can substantially influence the study’s results and conclusions. In our analysis, no significant differences were observed between the groups when considering only the first IMV period. However, when the first IMV period was combined with the second period (for patients requiring reintubation), the total ventilation time was twice as long in the Failure Group. This finding underscores the impact of extubation failure on total ventilation duration. A recent consensus has provided a clear definition for measuring IMV duration in pediatrics, recommending that all ventilation periods be included in the calculation.^7^

The incidence of extubation failure in our study was 5.2%, which is relatively low compared to rates reported in this literature. Previous studies have reported a wide variation in extubation failure rates, ranging from 6.2-19.3%.^10,16,17^ For example, Simonassi et al, in a study including 731 patients, reported a 19.3% incidence of extubation failure, which was associated with prolonged ventilation times in the Failure Group (231hs compared to 53hs in the Success Group). It is worth noting that the comparison of extubation failure rates across studies is facilitated by the consistent use of well-defined criteria for pediatric extubation failure. Currently, most studies define extubation failure as the need for reintubation within 48hs of extubation discontinuation, a standard definition widely adopted in the literature.^7,10,12,16,17^

Prolonged mechanical ventilation (MV) increases the risk of ventilator-associated pneumonia (VAP) airway trauma with subsequent edema and obstruction, muscle weakness, and significantly higher healthcare costs.^3,18-20^ Silva-Cruz et al. conducted a 5-years study and identified that MV durations exceeding 7 days were associated with an increased risk of reintubation.^21^ In contrast, our study showed a median MV duration of approximately 3 days, which is shorter compared to other studies reporting MV durations of 8-9 days.^17,22^

According to the literature, patients with pre-existing illnesses are at greater risk of complications during hospitalization. Several studies have shown that patients with neurological, cardiac, and respiratory comorbidities experience higher rates of reintubation than previously healthy patients.^10,17,23,24^ However, our study did not find a significant difference in the presence of pre-existing illnesses between extubation Success and Failure Groups.

In our study, the primary cause of extubation failure was UAO, identified in 67% of patients requiring reintubation. Upper airway obstruction has been long recognized in the literature as the leading cause of extubation failure in the pediatric population.^3,5,16,25,26^ Anatomical factors, such as the narrower and more compliant airways in children, predispose them to this compilation, resulting in airflow restriction, increased respiratory efforts, and higher work of breathing.^27^

Several studies have sought to identify predictive indices for stridor risk following extubation, such as the cuff leak test or air leak test.^20,28,29^ However, these methods have yielded inconclusive results in the pediatric population.

This study has some limitations. First, the retrospective nature of data collection and the lack of access to complete medical records resulted in missing data and the inability to analyze certain variables. Second, as a single-center study, the generalizability of the findings may be limited. Finally, the low incidence of extubation failure in our study may have restricted the statistical analysis of some results.

## CONCLUSION

Our study demonstrated that extubation failure doubled the total duration of mechanical ventilation compared to patients with successful extubation. These findings highlight the critical importance of daily patient assessments, guided by well-defined clinical criteria to ensure mechanical ventilation is discontinued precisely when the patient is clinically ready-neither prematurely nor unnecessarily delayed.
